# Draft Genome Sequence of Candida pseudohaemulonii Isolated from the Blood of a Neutropenic Patient

**DOI:** 10.1128/genomeA.00166-18

**Published:** 2018-04-05

**Authors:** Ratna Mohd Tap, Nur Amalina Kamarudin, Stephanie Jane Ginsapu, Ahmed Rafezzan Ahmed Bakri, Norazah Ahmad, Fairuz Amran, Matthias Sipiczki

**Affiliations:** a Mycology Laboratory, Bacteriology Unit, Infectious Diseases Research Centre, Institute for Medical Research, Jalan Pahang, Kuala Lumpur, Malaysia; b Microbiology Unit, Pathology Department, Queen Elizabeth Hospital, Kota Kinabalu, Sabah, Malaysia; c Department of Genetics and Applied Microbiology, University of Debrecen, Debrecen, Hungary

## Abstract

Candida pseudohaemulonii is phylogenetically close to the C. haemulonii complex and exhibits resistance to amphotericin B and azole agents. We report here the draft genome sequence of C. pseudohaemulonii UZ153_17 isolated from the blood culture of a neutropenic patient. The draft genome is 3,532,003,666 bp in length, with 579,838 reads, 130 contigs, and a G+C content of 47.15%.

## GENOME ANNOUNCEMENT

Candida pseudohaemulonii was first isolated in 2006 from the blood of a patient in Thailand (1). It is phylogenetically similar to C. haemulonii, which is also resistant to amphotericin B. C. pseudohaemulonii cannot be well differentiated by current commercial identification methods from its other related species, C. haemulonii, C. haemulonii var. vulnera, C. duobushaemulonii, and C. auris. Thus, a molecular barcode was proposed for fungal identification, namely, the internal transcribed spacer (ITS) region, the D1/D2 domain of the large subunit (LSU) region in the rRNA gene, and the matrix-assisted laser desorption ionization–time of flight mass spectrometry (MALDI-TOF MS) (2). In this article, we report the draft genome sequence of C. pseudohaemulonii UZ153_17 isolated from the blood culture of a neutropenic patient with short bowel syndrome.

DNA was extracted using the cetyltrimethylammonium bromide (CTAB) method (3), and a DNA library was prepared following the Illumina protocol using a TruSeq DNA LT sample prep kit (Illumina, San Diego, CA, USA). The genomic DNA was sequenced to a 451-fold depth of coverage using the Illumina HiSeq 2500 platform. From our analysis, the estimated genome size of C. pseudohaemulonii UZ153_17 is 14.74 Mb. The resulting draft genome’s total size is 3,532,003,666 bp, with an overall G+C content of 47.15%. The genome was assembled into 130 contigs using the SOAPdenovo version 2.04 assembler (4). All of the generated contigs were formed into 90 scaffolds, of which 47 are more than 1,000 bp in length, with a scaffold *N*_50_ size of 799,808 bp. The total size of all the scaffolds is 12,700,538 bp. Subsequent gene prediction analysis using MAKER2 (5) yielded a total of 5,540 predicted protein-coding genes with an average length of 1,492 bp. Genome annotation on the predicted genes was carried out by BLAST similarity searches against Swiss-Prot, a protein sequence database, with a total of 60.99% genes successfully annotated.

The genome is found to contain fluconazole and multidrug resistance genes. This finding is parallel with other studies that have shown that the isolate was resistant to amphotericin B and fluconazole (1, 2). The draft genome sequence of C. pseudohaemulonii will contribute to further investigations into the molecular mechanisms that lead to antifungal drug resistance.

### Accession number(s).

The nucleotide sequence of the C. pseudohaemulonii UZ153_17 genome has been deposited in DDBJ/EMBL/GenBank under accession no. PEJZ00000000.
